# Supplementary material for: Refractoriness of STING therapy is relieved by AKT inhibitor through effective vascular disruption in tumour
Source: Nat Commun. 2021 Jul 20;12:4405. doi: 10.1038/s41467-021-24603-w (PMC8292391; doi:10.1038/s41467-021-24603-w)
Supplement: Supplementary file 1 — Supplementary information [file 41467_2021_24603_MOESM1_ESM.pdf]

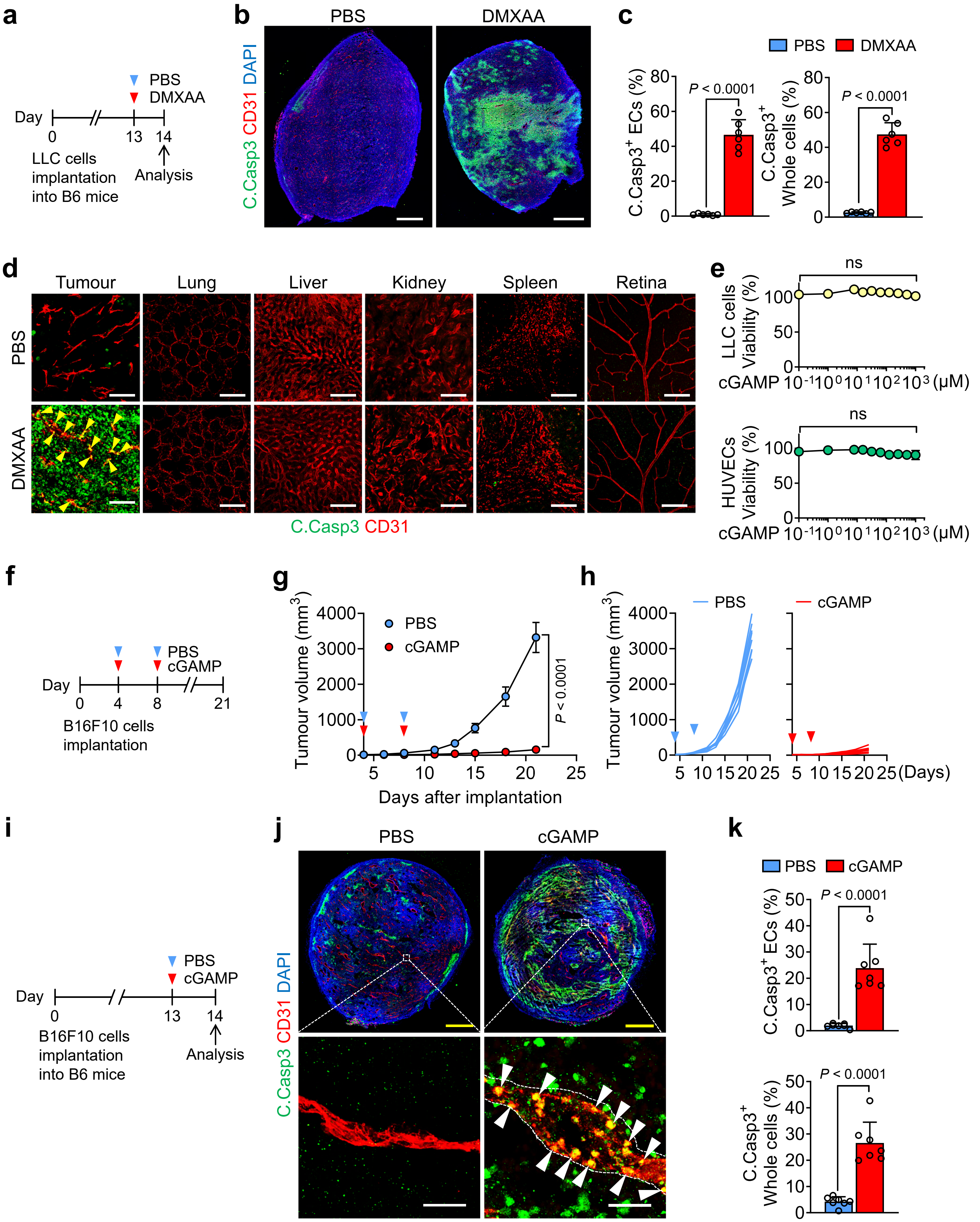
a
PBS
DMXAA
Day
0
13
14
LLC cells implantation into B6 mice
Analysis
b
PBS
DMXAA
C.Casp3 CD31 DAPI
c
PBS
DMXAA
C.Casp3+ ECs (%)
C.Casp3+ Whole cells (%)
P < 0.0001
d
Tumour
Lung
Liver
Kidney
Spleen
Retina
PBS
DMXAA
C.Casp3 CD31
e
ns
LLC cells Viability (%)
HUVECs Viability (%)
cGAMP
(μM)
f
PBS
cGAMP
Day
0
4
8
21
B16F10 cells implantation
g
PBS
cGAMP
Tumour volume (mm3)
Days after implantation
P < 0.0001
h
PBS
cGAMP
Tumour volume (mm3)
(Days)
i
PBS
cGAMP
Day
0
13
14
B16F10 cells implantation into B6 mice
Analysis
j
PBS
cGAMP
C.Casp3 CD31 DAPI
k
PBS
cGAMP
C.Casp3+ ECs (%)
C.Casp3+ Whole cells (%)
P < 0.0001

Supplementary Fig. 1. STING activation induces EC apoptosis exclusively in tumour

a–d, Diagram depicting generation of implanted LLC tumour in B6 mice and treatment schedule of intraperitoneal (i.p.) PBS or DMXAA and sampling at 24 h later. Representative images and comparison of apoptosis in tumour ECs and whole tumour cells (whole cells). Scale bars, 1.0 mm. n = 6 mice/group from two independent experiments. Vertical bars indicate mean ± SD. Representative images showing apoptotic ECs (yellow arrowheads) of tumour, lung, liver, kidney, spleen and retina in LLC tumour bearing mice. Scale bars, 100 μm. **e,** Viabilities of cultured LLC cells and HUVECs on concentrations of cGAMP for 24 h. Plots and bars indicate mean ± SD. **f-h,** Diagram depicting generation of implanted B16F10 melanoma in B6 mice and treatment schedule of i.t. PBS or cGAMP and sampling. Comparisons of B16F10 tumour growth. n = 8 mice/group from two independent experiments. Plots and bars indicate mean ± SD. **i–k,** Diagram depicting generation of implanted B16F10 melanoma in B6 mice and treatment schedule of i.t. PBS or cGAMP and sampling at 24 h later. Representative images and comparisons of apoptosis in tumour ECs and whole tumour cell. White arrowheads indicate apoptotic ECs. Scale bars, 1.0 mm (yellow bars) and 50 μm (white bars). n = 7 mice/group from two independent experiments. Vertical bars indicate mean ± SD. *P* values by two-tailed t-test. ns, not significant. Source data are provided as a Source Data file.

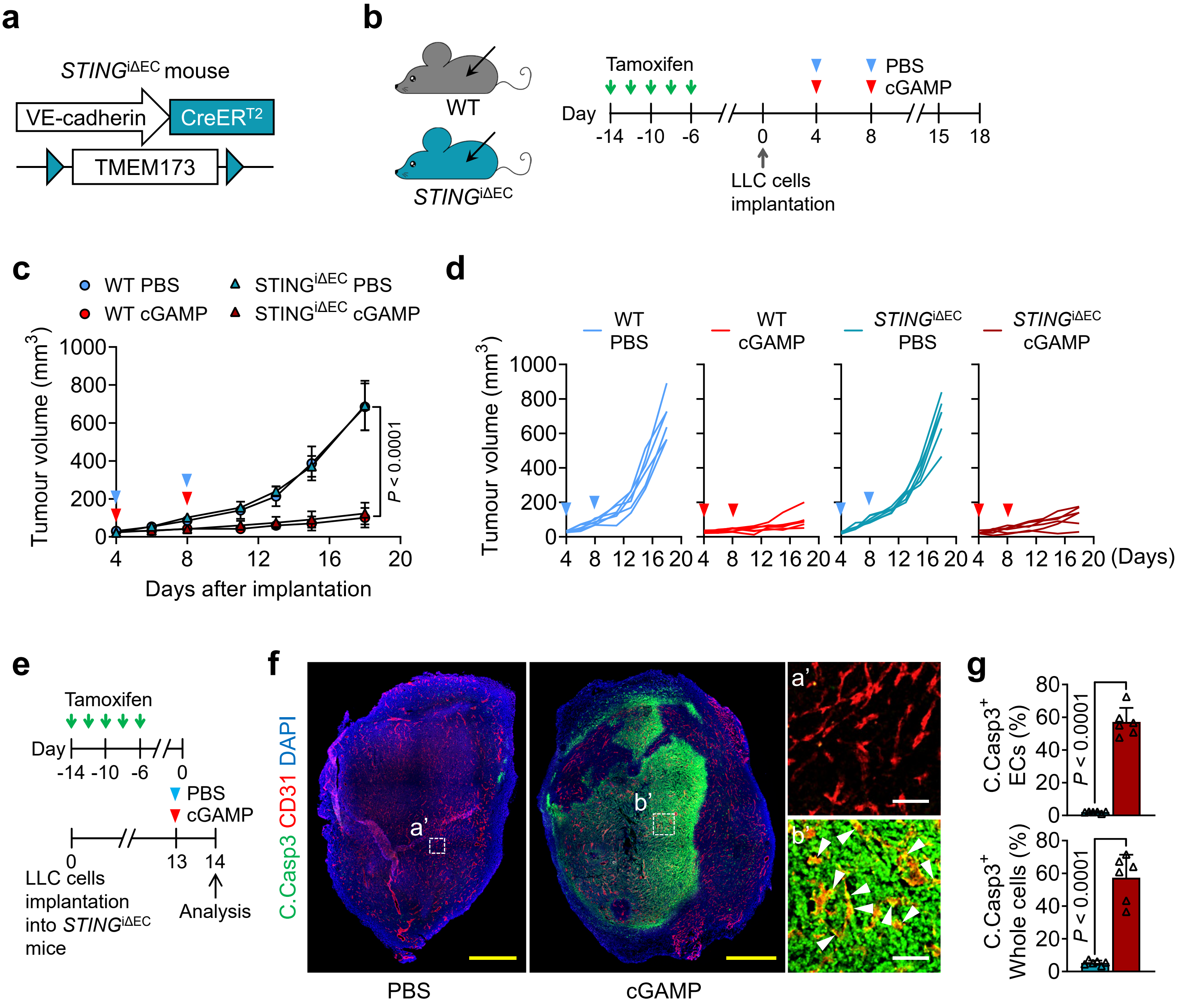

Supplementary Fig. 2. STING pathway of tumour EC is not required for cGAMP-induced apoptosis
a, Diagram for generation of EC-specific STING deleted (*STING*$^{i\Delta EC}$) mice. **b–d** Diagram depicting generation of implanted LLC tumour in *STING*$^{i\Delta EC}$ mice, i.p. administrations of tamoxifen (2 mg), and treatment schedule of i.t. PBS or cGAMP. Comparison of tumour growth. n = 6 mice/group from four independent experiments. Plots and vertical bars indicate mean ± SD. Plot indicates each individual tumour growth. *P* values by Welch's one-way ANOVA test followed by Dunnett's T3 test. **e–g,** Diagram depicting generation of implanted LLC tumour in *STING*$^{i\Delta EC}$ mice, i.p. administrations of tamoxifen (2 mg), treatment schedule of i.t. PBS or cGAMP, and sampling at 24 later. Representative images and comparisons of apoptosis in tumour ECs (white arrowheads) and whole tumour cells. Scale bars, 1.0 mm (yellow bars) and 100 µm (white bars). n = 6 mice/group from four experiments. Vertical bars indicate mean ± SD. *P* values by two-tailed t-test. Source data are provided as a Source Data file.

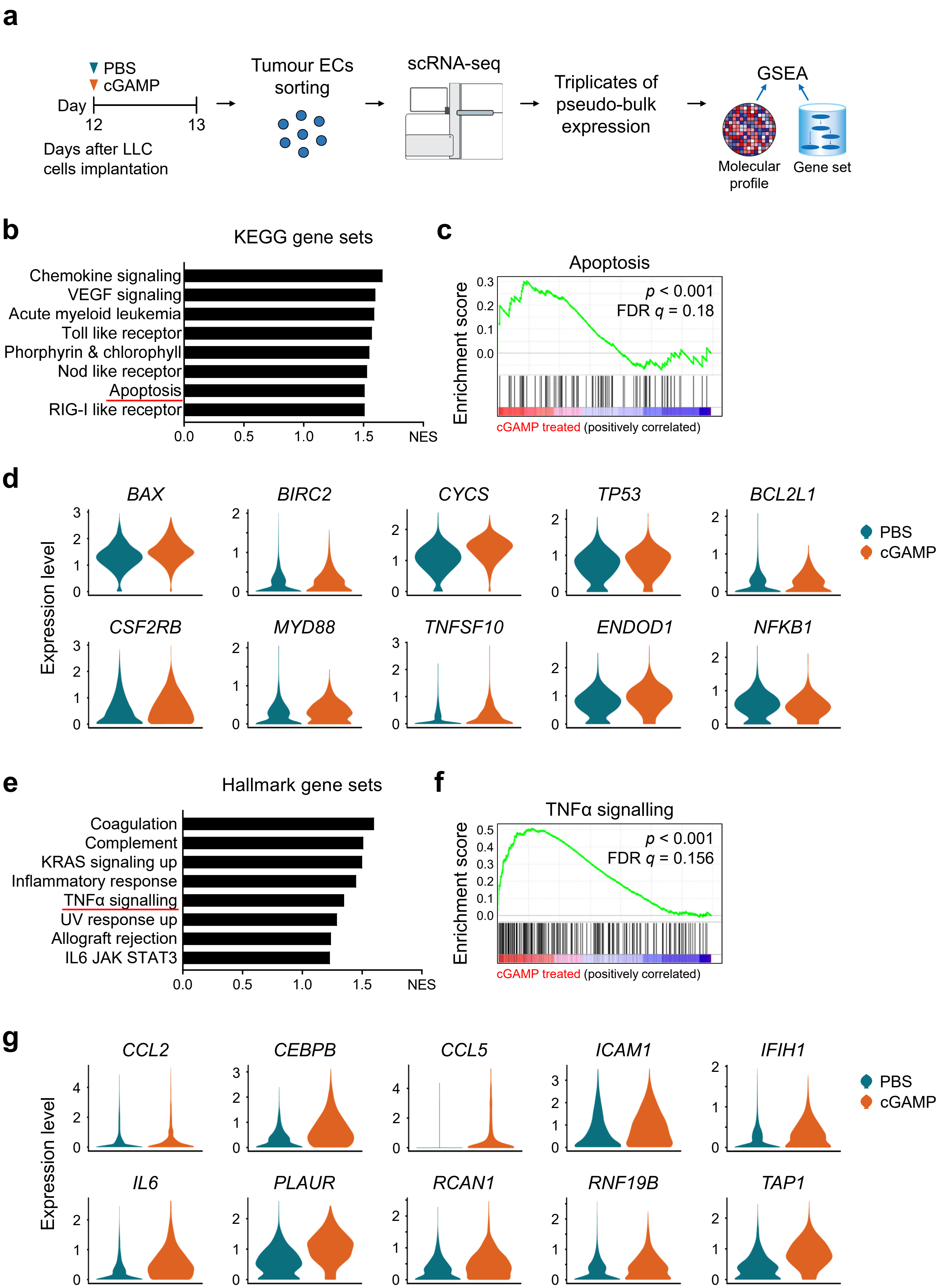
a
PBS
cGAMP
Day
12
13
Days after LLC cells implantation
Tumour ECs sorting
scRNA-seq
Triplicates of pseudo-bulk expression
GSEA
Molecular profile
Gene set
b
KEGG gene sets
Chemokine signaling
VEGF signaling
Acute myeloid leukemia
Toll like receptor
Phorphyrin & chlorophyll
Nod like receptor
Apoptosis
RIG-I like receptor
0.0
0.5
1.0
1.5
NES
c
Apoptosis
Enrichment score
p < 0.001
FDR q = 0.18
cGAMP treated (positively correlated)
d
BAX
BIRC2
CYCS
TP53
BCL2L1
CSF2RB
MYD88
TNFSF10
ENDOD1
NFKB1
Expression level
PBS
cGAMP
e
Hallmark gene sets
Coagulation
Complement
KRAS signaling up
Inflammatory response
TNFα signalling
UV response up
Allograft rejection
IL6 JAK STAT3
0.0
0.5
1.0
1.5
NES
f
TNFα signalling
Enrichment score
p < 0.001
FDR q = 0.156
cGAMP treated (positively correlated)
g
CCL2
CEBPB
CCL5
ICAM1
IFIH1
IL6
PLAUR
RCAN1
RNF19B
TAP1
Expression level
PBS
cGAMP

Supplementary Fig. 3. Genes related to apoptosis and TNFα signalling are enriched in tumour ECs by STING activation

a, Diagram depicting treatment of cGAMP and sampling of tumours 6 h later. Gene set enrichment analysis (GSEA) on pseudo-bulk expression generated from scRNA-seq to identify activated pathways by STING activation. **b, c,** Top 8 enriched pathways among KEGG gene sets in the cGAMP-treated tumour ECs. Mountain plot of apoptosis pathway. **d,** Violin plots depicting the normalized expression levels of genes showing the enrichment of apoptosis signalling in the cGAMP-treated tumour ECs. **e, f,** Top 8 enriched pathways among Hallmark gene sets in the cGAMP-treated tumour ECs. Mountain plot of TNFα signalling. **g,** Violin plots depicting the normalized expression levels of genes showing the enrichment of TNFα signalling in the cGAMP-treated tumour ECs. FDR, false discovery rate; NES, normalized enrichment score. Nominal *p* value was shown in **c** and **f**.

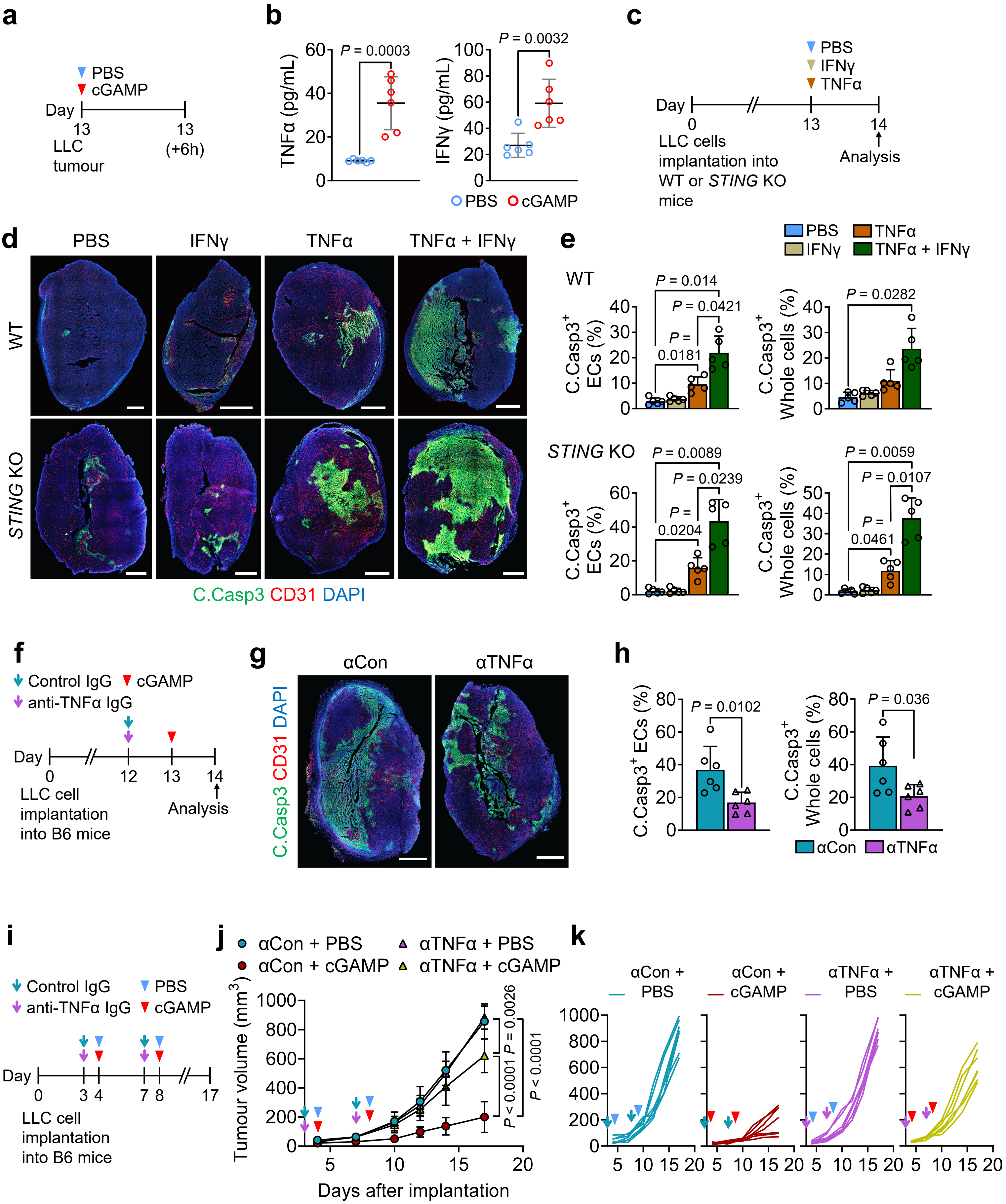

a
PBS
cGAMP
Day 13 LLC tumour
13 (+6h)
b
TNFα (pg/mL)
P = 0.0003
IFNγ (pg/mL)
P = 0.0032
PBS cGAMP
c
PBS
IFNγ
TNFα
Day 0 LLC cells implantation into WT or STING KO mice
13
14 Analysis
d
PBS IFNγ TNFα TNFα + IFNγ
WT
STING KO
C.Casp3 CD31 DAPI
e
PBS TNFα IFNγ TNFα + IFNγ
WT
C.Casp3+ ECs (%)
P = 0.014
P = 0.0421
P = 0.0181
C.Casp3+ Whole cells (%)
P = 0.0282
STING KO
P = 0.0089
P = 0.0239
P = 0.0204
P = 0.0059
P = 0.0107
P = 0.0461
f
Control IgG
cGAMP
anti-TNFα IgG
Day 0 LLC cell implantation into B6 mice
12 13 14 Analysis
g
αCon αTNFα
C.Casp3 CD31 DAPI
h
C.Casp3+ ECs (%)
P = 0.0102
C.Casp3+ Whole cells (%)
P = 0.036
αCon αTNFα
i
Control IgG PBS
anti-TNFα IgG cGAMP
Day 0 3 4 7 8 17
LLC cell implantation into B6 mice
j
αCon + PBS
αCon + cGAMP
αTNFα + PBS
αTNFα + cGAMP
Tumour volume (mm3)
Days after implantation
P = 0.0026
P < 0.0001
P < 0.0001
k
αCon + PBS
αCon + cGAMP
αTNFα + PBS
αTNFα + cGAMP

Supplementary Fig. 4. Activation of TNFα-TNFR1 following cGAMP-treatment plays a critical role in tumour EC Apoptosis and anti-tumour effect

a, b, Diagram depicting treatment and sampling at 6 h later in the LLC implantation tumour mice. Comparison of TNFα and IFNγ in tumour lysates between i.t. PBS- and cGAMP-treated tumours. n = 6 mice/group from two independent experiments. Horizontal bars indicate mean ± SD. **c–e,** LLC tumours in WT and *STING* KO mice were analysed 24 h after i.t. injection of TNFα (210 ng/70 µl), IFNγ (350 ng/70 µl) or combination of TNFα and IFNγ in 70 µl. Representative images and comparisons of apoptosis in tumour ECs and whole tumour cells (whole cells). n = 5 mice/group from four independent experiments. Vertical bars indicate mean ± SD. **f–h,** Diagram depicting treatment and sampling in LLC implanted tumour mice. Representative images and comparisons of apoptosis in tumour ECs and whole tumour cells following each treatment. Scale bars, 1.0 mm. n = 6 mice/group from two independent experiments. Vertical bars indicate mean ± SD. **i–k,** Diagram depicting treatment schedule in LLC implanted tumour mice. Comparisons of tumour growths. n = 7 mice/group from four independent experiments. Plots and bars indicate mean ± SD. Plot indicates each individual tumour growth. *P* values by two-tailed t-test (**b**, **h**) or Welch's one-way ANOVA test followed by Dunnett's T3 test (**e**, **j**). ns, not significant. Source data are provided as a Source Data file.

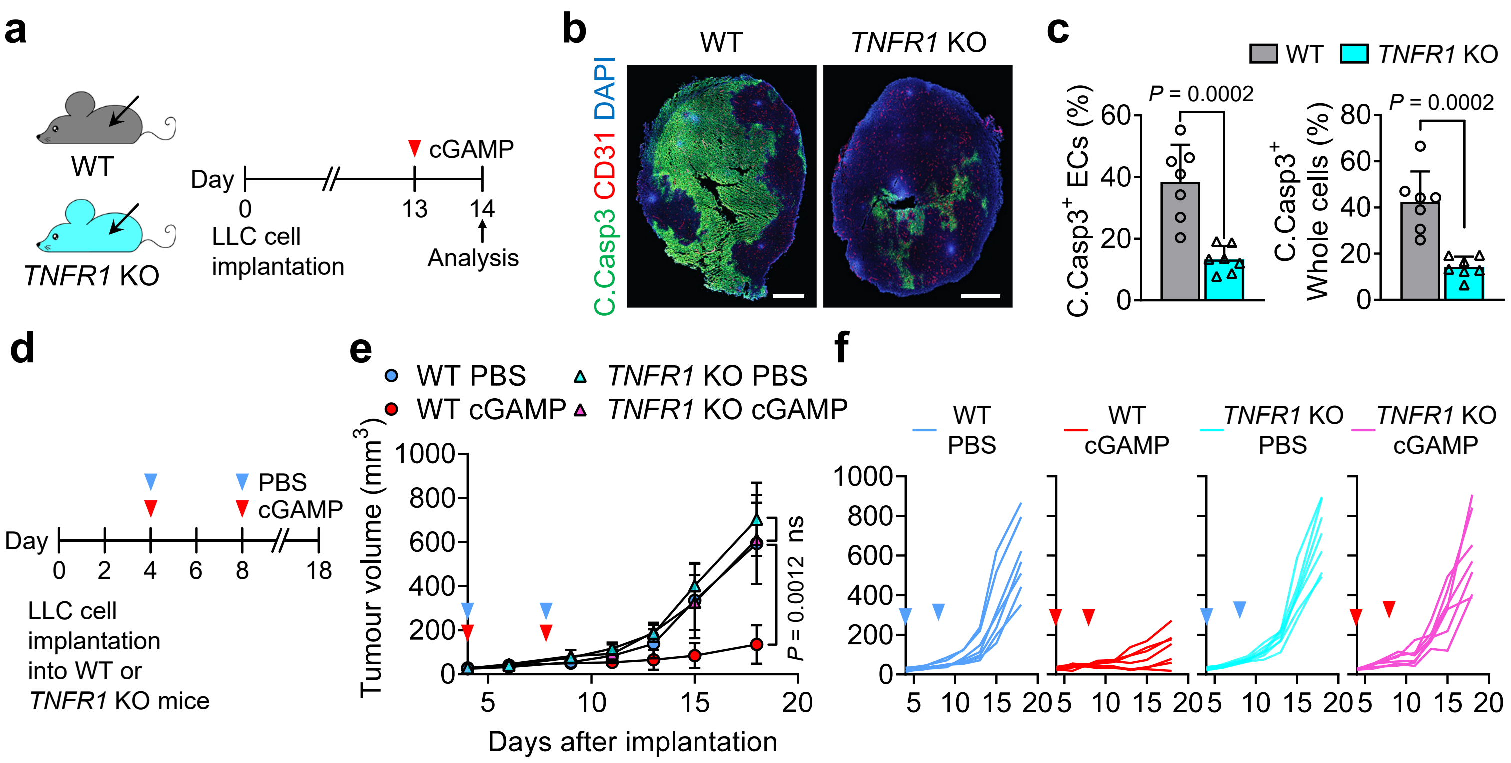

Supplementary Fig. 5. cGAMP-induced tumour EC apoptosis and anti-tumour effects are hampered in LLC tumours of *TNFR1* KO mice

a–c, Diagram depicting cGAMP treatment and tumour sampling at 24 h later in WT and *TNFR1* KO mice. Representative images and comparisons of apoptosis in tumour ECs and whole tumour cells (whole cells). n = 7 mice/group from two independent experiments. Vertical bars indicate mean ± SD. **d–f,** Diagram depicting treatment schedule and tumour growth in WT or *TNFR1* KO mice. Comparison of LLC tumour growth. n = 7 mice/group from four independent experiments. Plots and bars indicate mean ± SD. Plot indicates each individual tumour growth. *P* values by two-tailed t-test (**c**) or Welch's one-way ANOVA test followed by Dunnett's T3 test (**e**). ns, not significant. Source data are provided as a Source Data file.

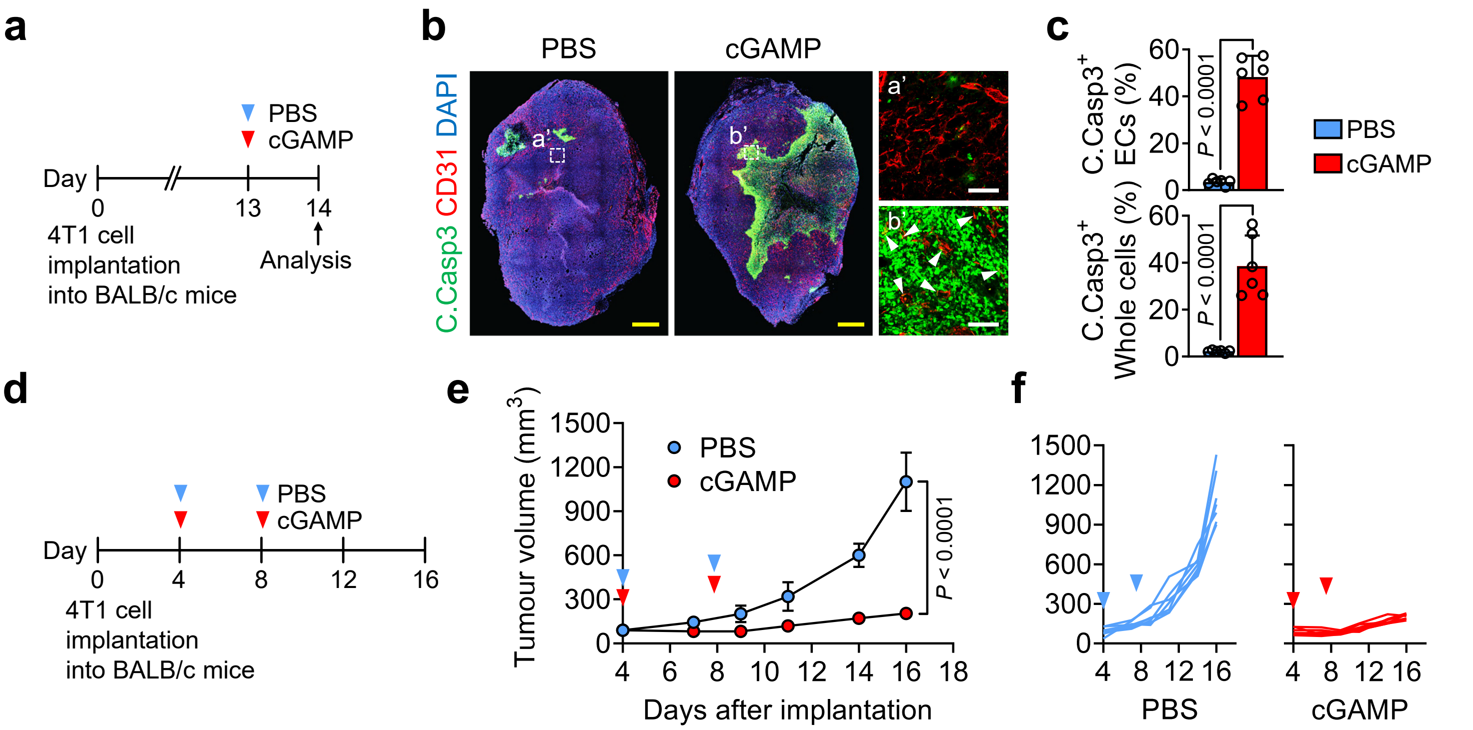

Supplementary Fig. 6. cGAMP induces tumour EC apoptosis and strong anti-tumour effect in 4T1 breast tumours

a–c, Diagram depicting generation of orthotopic implanted 4T1 breast tumours in BALB/c mice, i.t. PBS or cGAMP treatment, and sampling 24 h later. Representative images and comparisons of apoptosis in tumour ECs (white arrowheads) and whole tumour cells (whole cells). Scale bars, 1.0 mm (yellow bars) and 100 μm (white bars). n = 6 mice/group from two independent experiments. Vertical bars indicate mean ± SD. **d–f,** Diagram depicting treatment schedule in orthotopic implanted 4T1 breast tumour mice. Comparison of tumour growth. n = 7 mice/group from two independent experiments. Plots and bars indicate mean ± SD. Plot indicates each individual tumour growth.

P values by two-tailed t-test (**c**, **e**) . Source data are provided as a Source Data file.

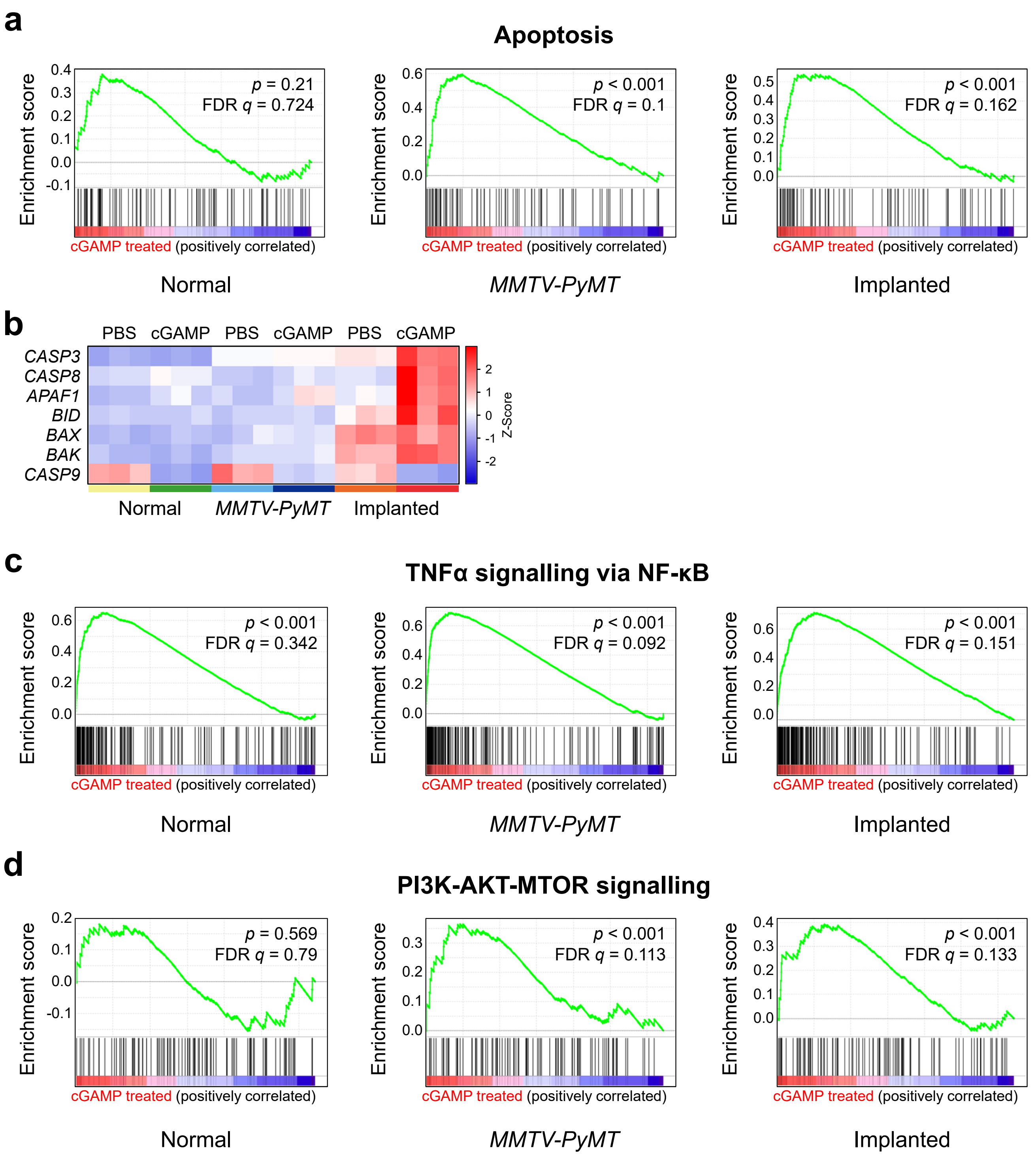

Supplementary Fig. 7. STING activation leads to enrichment of the genes associated with apoptosis and AKT signalling pathway in ECs or tumour ECs

a-d, ECs were obtained from mammary tissues of normal female mice, while tumour ECs were obtained from the breast tumours of *MMTV-PyMT* mice and its implantation model mice, and performed bulk RNA-seq. **a,** GSEA for apoptosis pathway in the ECs treated with cGAMP *versus* PBS. **b,** Heatmap of the RNA-seq data performed on pro-apoptotic genes. **c, d,** GSEA for TNFα signalling via NF-κB and PI3K-AKT-mTOR signalling pathways in the ECs treated with cGAMP *versus* PBS. FDR, false discovery rate. Nominal *p* value was shown in **a, c,** and **d**.

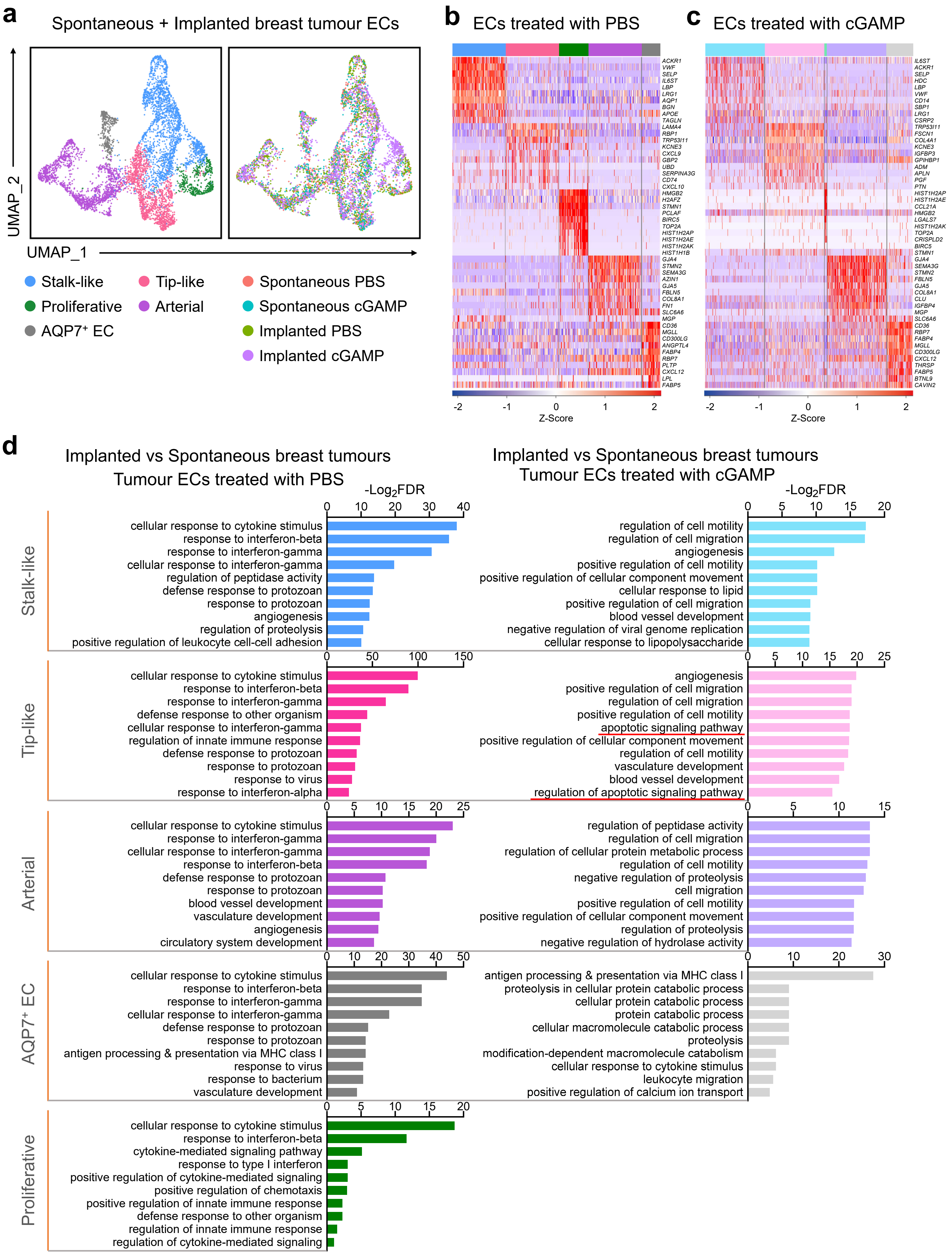

a
Spontaneous + Implanted breast tumour ECs
UMAP_2
UMAP_1
Stalk-like
Tip-like
Proliferative
Arterial
AQP7⁺ EC
Spontaneous PBS
Spontaneous cGAMP
Implanted PBS
Implanted cGAMP
b
ECs treated with PBS
ACKR1 VWF SELP IL6ST LBP LRG1 AQP1 BGN APOE TAGLN LAMA4 RBP1 TRP53I11 KCNE3 CXCL9 GBP2 UBD SERPINA3G CD74 CXCL10 HMGB2 H2AFZ STMN1 PCLAF BIRC5 TOP2A HIST1H2AP HIST1H2AE HIST1H2AK HIST1H1B GJA4 STMN2 SEMA3G AZIN1 GJA5 FBLN5 COL8A1 FN1 SLC6A6 MGP CD36 MGLL CD300LG ANGPTL4 FABP4 RBP7 PLTP CXCL12 LPL FABP5
-2 -1 0 1 2
Z-Score
c
ECs treated with cGAMP
IL6ST ACKR1 SELP HDC LBP VWF CD14 SBP1 LRG1 CSRP2 TRP53I11 FSCN1 COL4A1 KCNE3 IGFBP3 GPIHBP1 ADM APLN PGF PTN HIST1H2AP HIST1H2AE CCL21A HMGB2 LGALS7 HIST1H2AK TOP2A CRISPLD2 BIRC5 STMN1 GJA4 SEMA3G STMN2 FBLN5 GJA5 COL8A1 CLU IGFBP4 MGP SLC6A6 CD36 RBP7 FABP4 MGLL CD300LG CXCL12 THRSP FABP5 BTNL9 CAVIN2
-2 -1 0 1 2
Z-Score
d
Implanted vs Spontaneous breast tumours
Tumour ECs treated with PBS
-Log2FDR
Stalk-like
0 10 20 30 40
cellular response to cytokine stimulus
response to interferon-beta
response to interferon-gamma
cellular response to interferon-gamma
regulation of peptidase activity
defense response to protozoan
response to protozoan
angiogenesis
regulation of proteolysis
positive regulation of leukocyte cell-cell adhesion
Tip-like
0 50 100 150
cellular response to cytokine stimulus
response to interferon-beta
response to interferon-gamma
defense response to other organism
cellular response to interferon-gamma
regulation of innate immune response
defense response to protozoan
response to protozoan
response to virus
response to interferon-alpha
Arterial
0 5 10 15 20 25
cellular response to cytokine stimulus
response to interferon-gamma
cellular response to interferon-gamma
response to interferon-beta
defense response to protozoan
response to protozoan
blood vessel development
vasculature development
angiogenesis
circulatory system development
AQP7⁺ EC
0 10 20 30 40 50
cellular response to cytokine stimulus
response to interferon-beta
response to interferon-gamma
cellular response to interferon-gamma
defense response to protozoan
response to protozoan
antigen processing & presentation via MHC class I
response to virus
response to bacterium
vasculature development
Proliferative
0 5 10 15 20
cellular response to cytokine stimulus
response to interferon-beta
cytokine-mediated signaling pathway
response to type I interferon
positive regulation of cytokine-mediated signaling
positive regulation of chemotaxis
positive regulation of innate immune response
defense response to other organism
regulation of innate immune response
regulation of cytokine-mediated signaling
Implanted vs Spontaneous breast tumours
Tumour ECs treated with cGAMP
-Log2FDR
0 5 10 15 20
regulation of cell motility
regulation of cell migration
angiogenesis
positive regulation of cell motility
positive regulation of cellular component movement
cellular response to lipid
positive regulation of cell migration
blood vessel development
negative regulation of viral genome replication
cellular response to lipopolysaccharide
0 5 10 15 20 25
angiogenesis
positive regulation of cell migration
regulation of cell migration
positive regulation of cell motility
apoptotic signaling pathway
positive regulation of cellular component movement
regulation of cell motility
vasculature development
blood vessel development
regulation of apoptotic signaling pathway
0 5 10 15
regulation of peptidase activity
regulation of cell migration
regulation of cellular protein metabolic process
regulation of cell motility
negative regulation of proteolysis
cell migration
positive regulation of cell motility
positive regulation of cellular component movement
regulation of proteolysis
negative regulation of hydrolase activity
0 10 20 30
antigen processing & presentation via MHC class I
proteolysis in cellular protein catabolic process
cellular protein catabolic process
protein catabolic process
cellular macromolecule catabolic process
proteolysis
modification-dependent macromolecule catabolism
cellular response to cytokine stimulus
leukocyte migration
positive regulation of calcium ion transport

Supplementary Fig. 8. Implanted breast tumour ECs are more susceptible to cGAMP-induced apoptosis and more inflammatory compared with spontaneous breast tumour ECs

a, UMAP plots integrating all tumour ECs of spontaneous and implanted breast tumours treated with PBS or cGAMP. **b, c,** Heatmap visualizing distinctive expression profiles in PBS- or cGAMP-treated tumour ECs derived from both spontaneous and implanted tumours. n = 2 mice/each group. Scaled expression levels of top ten differentially expressed genes for indicated clusters are shown. **d**, Gene ontology (GO) enrichment analyses on highly expressed genes in implanted tumour ECs compared with spontaneous tumour ECs. Left and right panels represent GO enrichment analysis results on PBS- and cGAMP-treated tumour ECs. n = 2 mice/each group.

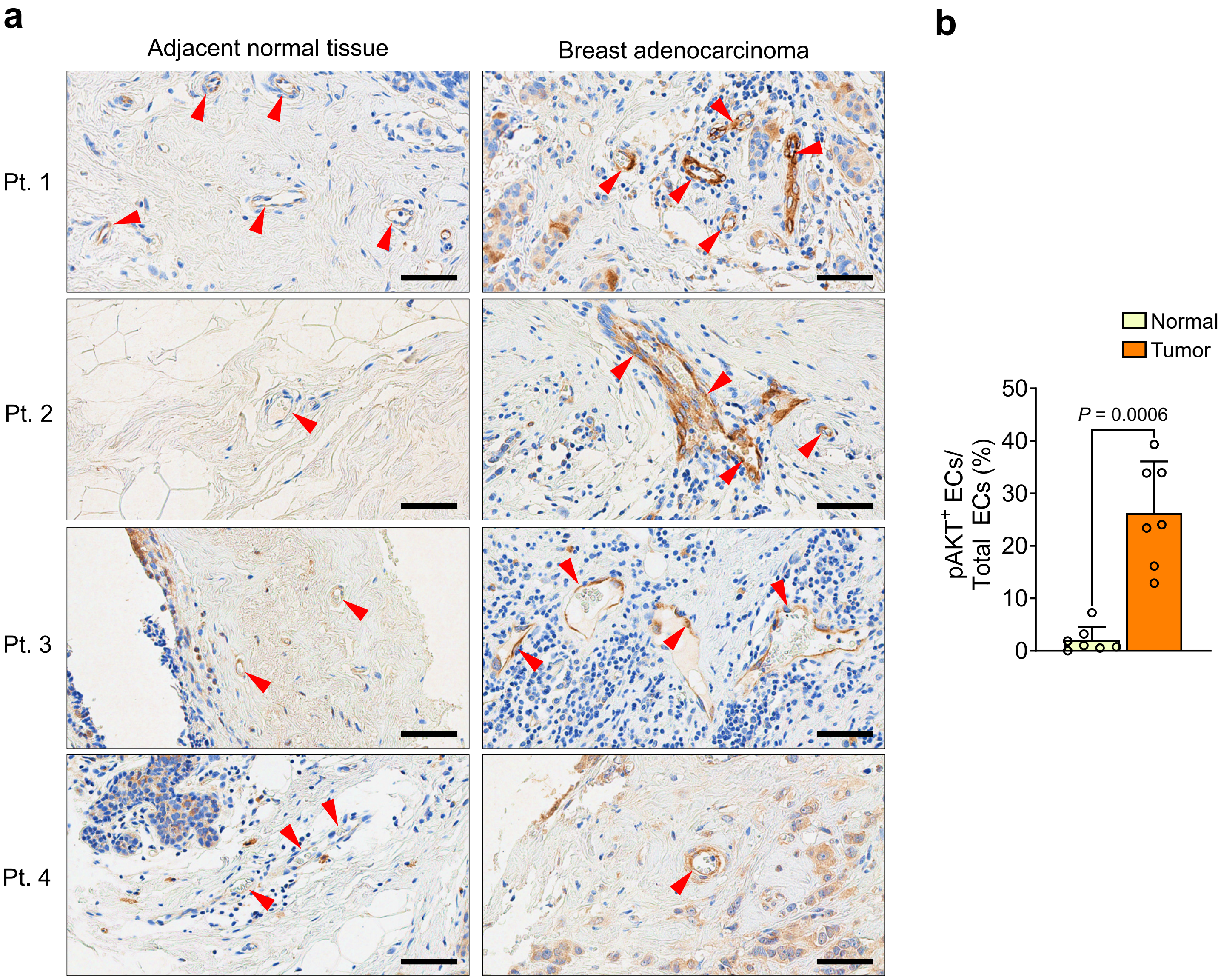

Supplementary Fig. 9. Tumour ECs of human breast carcinomas have stronger AKT phosphorylation at S473 (pAKT) compared with those of their adjacent normal tissues

a, Representative images of pAKT in the ECs of sectioned breast adenocarcinoma tissues from 4 patients (Pt.). Red arrows indicate blood vessels. Scale bars, 60 μm. **b**, Comparison of $pAKT^{+}$ ECs/total ECs. n = 7/group from two independent experiments. *P* values by two-tailed t-test. Vertical bars indicate mean ± SD. Source data are provided as a Source Data file.

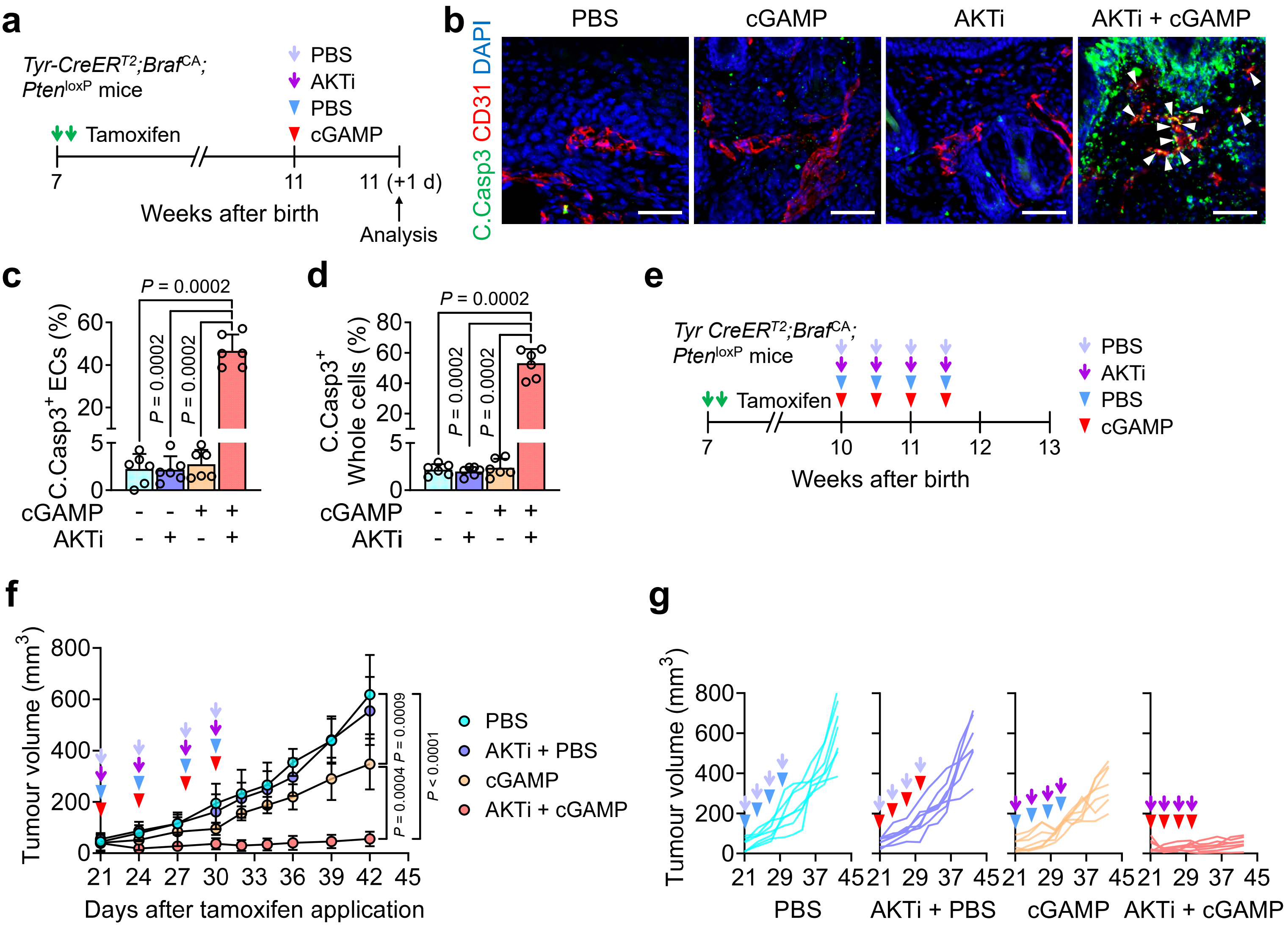

Supplementary Fig. 10. Combined treatment of cGAMP and AKTi induces tumour EC apoptosis and potent anti-tumour effect in spontaneous melanoma

a–d, Diagram depicting tamoxifen swabbing, treatment and tumour sampling in *Tyr-CreER*^{T2}*;Braf*^{CA}*;Pten*^{loxP} spontaneous melanoma mice. Representative images of tumour vessels and apoptosis. White arrowheads indicate tumour EC apoptosis. Scale bars, 50 μm. Comparisons of apoptosis in tumour ECs and whole tumour cells (whole cells). n = 6 mice/group from four independent experiments. Vertical bars indicate mean ± SD. **e–g**, Diagram depicting tamoxifen swabbing and treatment schedule. Comparisons of tumour growths. n = 7 mice/group from four independent experiments. Plots and bars indicate mean ± SD. Plot indicates each individual tumour growth. *P* values by Welch's one-way ANOVA test followed by Dunnett's T3 test (**c**, **d**, **f**). ns, not significant. Source data are provided as a Source Data file.

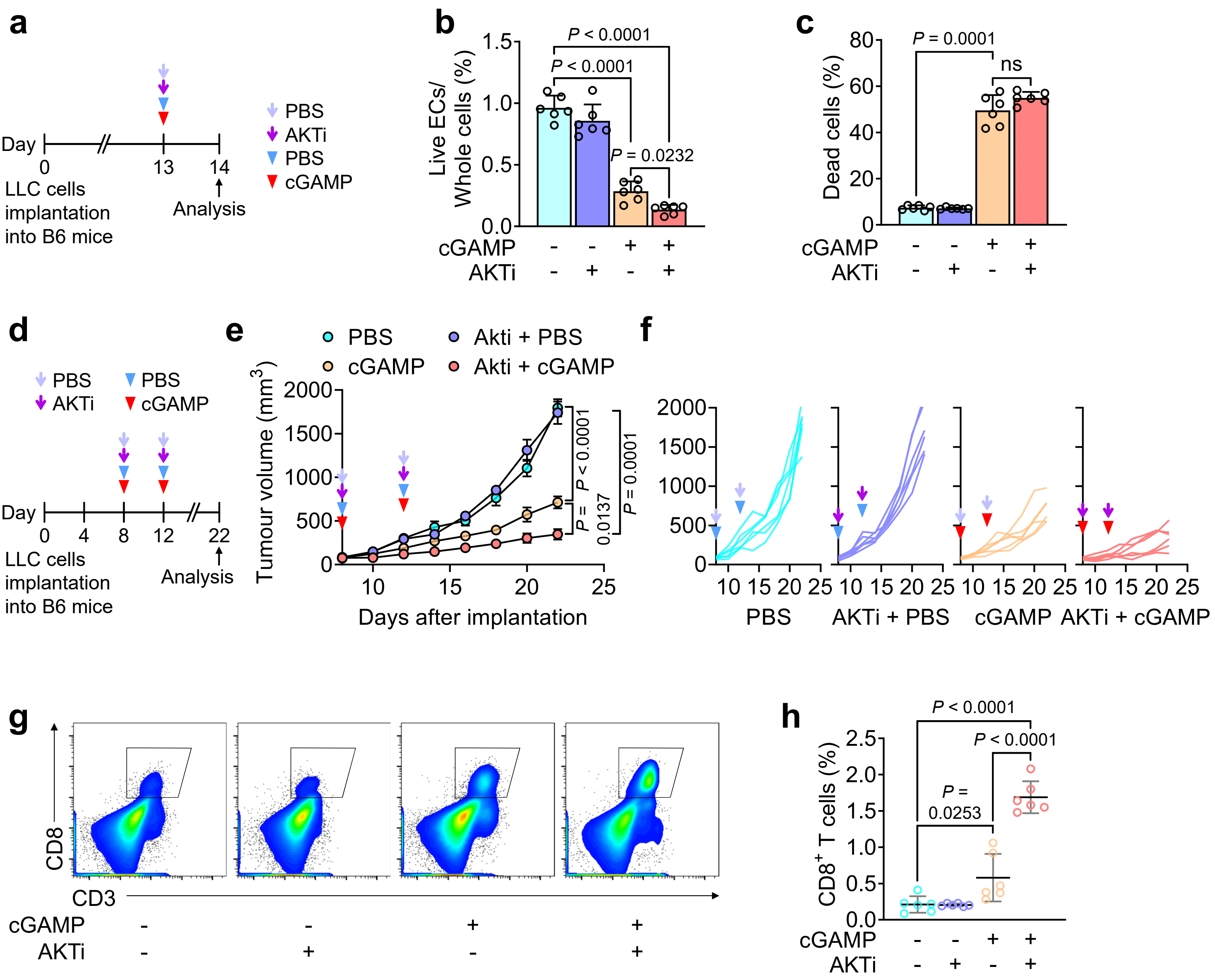

Supplementary Fig. 11. AKT inhibitor potentiates anti-tumour effect of cGAMP in LLC tumour

a–c, LLC tumour was treated with PBS or cGAMP with or without AKTi, and sampling at 24 h later. Comparisons of populations of live ECs (per whole cells) and dead cells in LLC tumours. n = 6 mice/group from four independent experiments. Vertical bars indicate mean ± SD. **d–h,** LLC tumours were treated with PBS or cGAMP with or without AKTi twice with 4 days interval. Comparisons of tumour growths. n = 6 mice/group from four independent experiments. Plots and bars indicate mean ± SD. Plot indicates each individual tumour growth. Representative flow cytometry plots and comparisons of CD8⁺ T cell infiltration in LLC tumour following each treatment. n = 6 mice/group from four independent experiments. Horizontal bars indicate mean ± SD. *P* values by Welch's one-way ANOVA test followed by Dunnett's T3 test (**b**, **c**, **e**) or one-way ANOVA test followed by Tukey's test (**h**). ns, not significant. Source data are provided as a Source Data file.

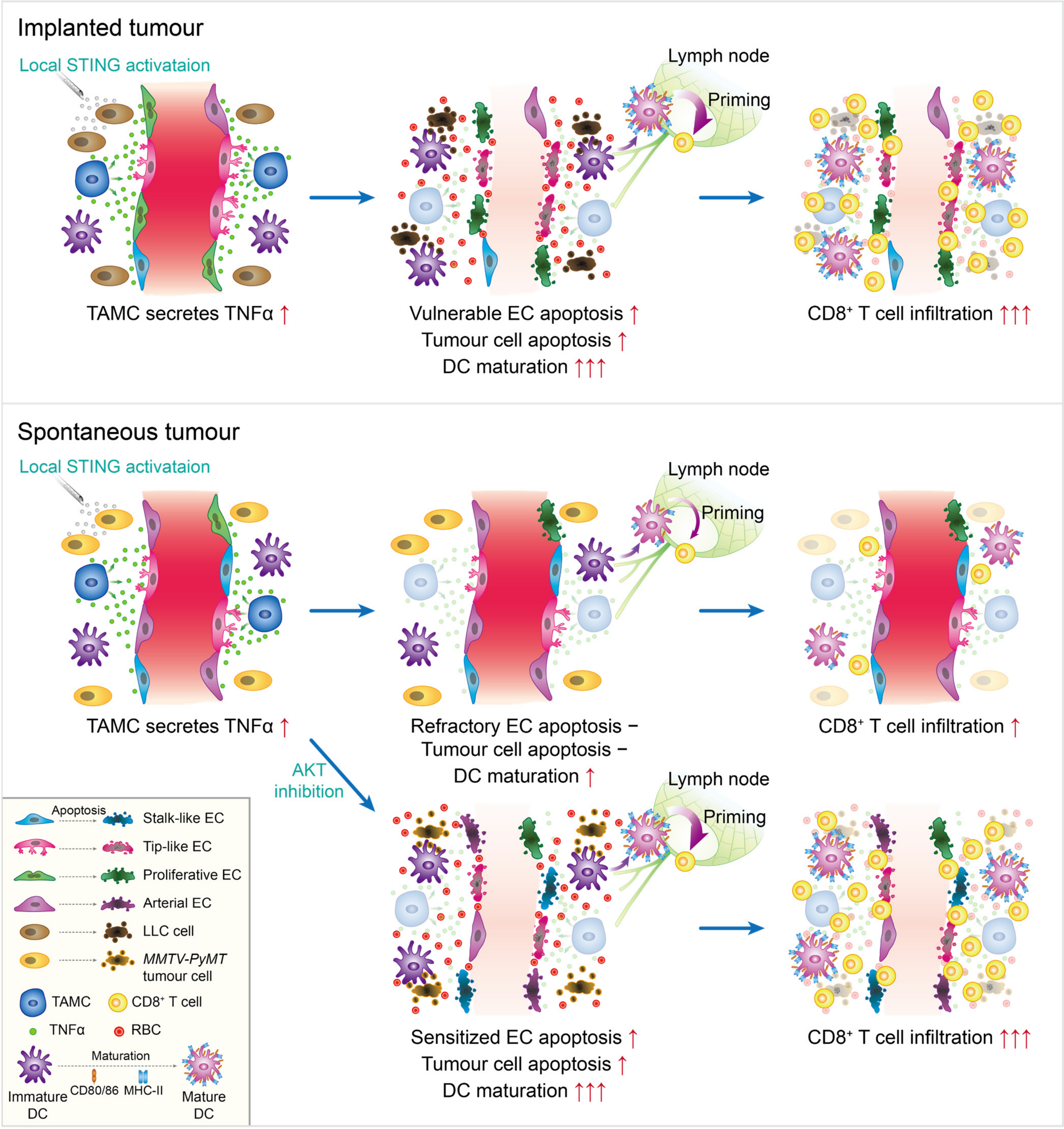

Supplementary Fig. 12. Schematic diagram for the proposed mechanism of STING activation–induced tumour EC apoptosis and enforcement of anti-tumour effect via AKTi in refractory spontaneous tumours In tumour microenvironment, STING activation triggers TNFα production from TAMCs, leading to extensive tumour EC apoptosis through TNFα-TNFR1. The resulting whole tumour cell apoptosis renders a massive release of tumour antigens for priming and activation of CD8$^+$ T cells in implantation tumours. However, in spontaneous tumours, apoptosis of tumour ECs and whole tumour cells is truncated, resulting in incompetent adaptive immune activation. Nevertheless, AKTi could sensitize the resistant tumour ECs to STING activation-induced apoptosis. Accordingly, combined treatment with AKTi and cGAMP induces extensive tumour EC apoptosis followed by massive tumour cell apoptosis to establish sufficient anti-tumour immunity in spontaneous tumours.

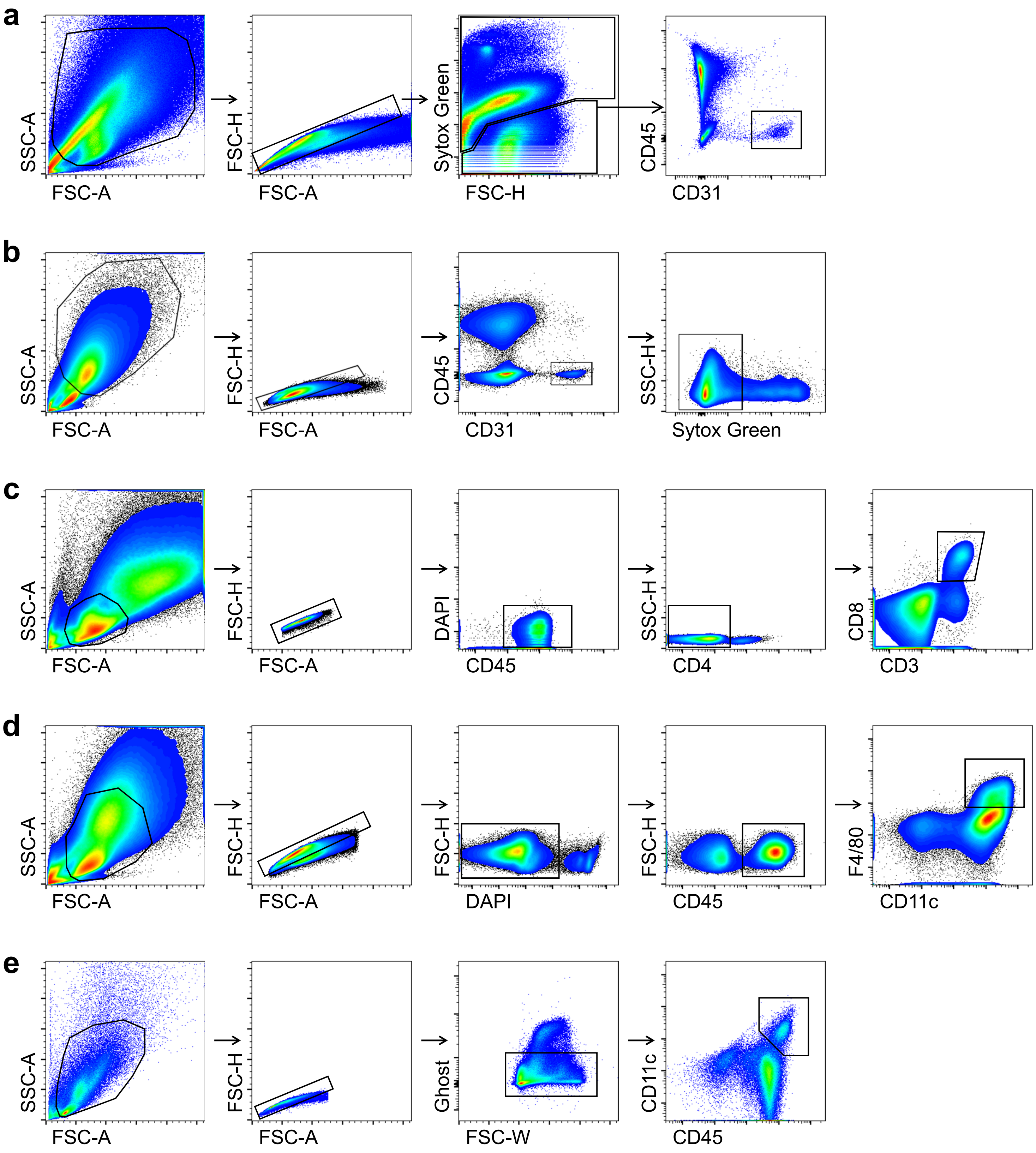

Supplementary Fig. 13. Sequential gating strategies used for flow cytometry or cell sorting

a, Gating strategy to analyse live ECs and whole dead cells in Fig. 1h, 1j, 2g and 2i. **b**, Gating strategy for sorting live ECs in Fig. 4, 7 and 8. **c**, Gating strategy to analyse $CD8^+$ T cells in Fig. 5j and 10k and Supplementary Fig. 11g. **d**, Gating strategy to analyse tumour associated macrophages in Fig. 5b. **e**, Gating strategy to analyse DCs of TDLN in Fig. 10e-g.

Supplementary Table 1. Quality metrics of scRNA-seq

Sample		NUM Cell	Mean nGene	Mean nUMI	Final NUM Cell
**Tumour ECs from LLC tumours**	PBS	4212	4506	33518	2517
	STING	2362	4921	40815	1340
**Tumour ECs from breast tumours**	MP1	5256	2573	14595	768
	MP2	3909	2700	17562	454
	MS1	4574	2301	14960	460
	MS2	4690	2292	13536	486
	IP1	2740	2642	16164	773
	IP2	4536	2740	16979	910
	IS1	9478	1607	7304	1454
	IS2	3239	2333	13048	490

NUM, number; nGene, number of genes detected in each cell; nUMI, number of unique molecular identifier; MP, MMTV-PyMT mice breast tumour treated with i.t. PBS; MS, MMTV-PyMT mice breast tumour treated with i.t. cGAMP; IP, implanted breast tumour treated with i.t. PBS; IS, implanted breast tumour treated with i.t. cGAMP.
